# P20. Lack of T cell exhaustion in acute myeloid leukaemia

**DOI:** 10.1186/2051-1426-2-S2-P11

**Published:** 2014-03-12

**Authors:** FM Schnorfeil, FS Lichtenegger, K Emmerig, M Schlüter, R Draenert, W Hiddemann, M Subklewe

**Affiliations:** 1 Helmholtz Institute Munich, Clinical Cooperation Group Immunotherapy, Munich, Germany; 2 Klinikum der Universität München, Department of Internal Medicine IV, Munich, Germany; 3 Klinikum der Universität München, Department of Internal Medicine III, Munich, Germany

## 

The prognosis of acute myeloid leukemia (AML), particularly when associated with adverse chromosomal or molecular aberrations, is poor due to a high relapse rate after induction chemotherapy. Postremission therapy for elimination of minimal residual disease remains a major challenge. Immunotherapeutic strategies aim at the stimulation of AML-specific immunity, especially of CD8^+^ T cells. However, the functionality of these cells in AML patients is not well described. T cell exhaustion has been suggested to contribute to immune evasion in various solid and haematological malignancies. Primarily demonstrated in chronic viral infections, exhausted T cells are characterised by an increased expression of several inhibitory molecules, reduced proliferation and an impaired capability of cytokine secretion and cytotoxicity.

To characterise T cell exhaustion in AML, we assessed the phenotype and effector function of CD8^+^ and CD4^+^ T cells by flow cytometry. T cells from patients at primary diagnosis, with refractory disease, at relapse and at relapse after allogeneic stem cell transplantation (alloSCT) were analysed for surface expression of CD244, CD160, PD-1, TIM-3 and LAG-3. T cell proliferation and production of the cytokines IFN-γ, TNF-α and IL-2 were measured in response to different stimuli. Results were compared to healthy controls (HCs), while untreated HIV-infected patients served as positive controls for an exhausted T cell state.

In HIV-infected patients, we observed a pronounced upregulation of the inhibitory molecules CD244, CD160 and PD-1 on CD4^+^ and CD8^+^ T cells as well as globally impaired cytokine production, clearly indicating T cell exhaustion. In contrast, T cells from AML patients showed an expression pattern of inhibitory surface molecules that was similar to T cells from age-matched HCs. AML patients with a relapse after alloSCT, however, showed remarkably high PD-1 expression on CD4^+^ and CD8^+^ T cells, accompanied by a shift from naive to memory T cells. Functionally, no defect in T cell proliferation in any of the AML patient cohorts was detected. Of note, however, we observed a 2-fold decrease in IFN-γ production by CD4^+^ T cells exclusively in patients at primary diagnosis.

Thus, T cells of AML patients are fully functional. Immunotherapies that aim at eliciting tumour-specific immune responses, e.g. dendritic cell based vaccines, may therefore be particularly suited for AML treatment.

